# Synthesis of Carbon Nanotubes of Few Walls Using Aliphatic Alcohols as a Carbon Source

**DOI:** 10.3390/ma6062534

**Published:** 2013-06-20

**Authors:** Elsa G. Ordoñez-Casanova, Manuel Román-Aguirre, Alfredo Aguilar-Elguezabal, Francisco Espinosa-Magaña

**Affiliations:** Laboratorio Nacional de Nanotecnología, Centro de Investigación en Materiales Avanzados, S.C., Miguel de Cervantes 120, Complejo Industrial Chihuahua, 31109 Chihuahua, Chih., Mexico; E-Mails: gabriela.ordonez@cimav.edu.mx (E.G.O.-C.); manuel.roman@cimav.edu.mx (M.R.-A.); alfredo.aguilar@cimav.edu.mx (A.A.-E.)

**Keywords:** carbon nanotubes (CNT), aliphatic alcohols, spray pyrolysis

## Abstract

Carbon nanotubes with single and few walls are highly appreciated for their technological applications, regardless of the limited availability due to their high production cost. In this paper we present an alternative process that can lead to lowering the manufacturing cost of CNTs of only few walls by means of the use of the spray pyrolysis technique. For this purpose, ferrocene is utilized as a catalyst and aliphatic alcohols (methanol, ethanol, propanol or butanol) as the carbon source. The characterization of CNTs was performed by scanning electron microscopy (SEM) and transmission electron microscopy (TEM). The study of the synthesized carbon nanotubes (CNTs) show important differences in the number of layers that constitute the nanotubes, the diameter length, the quantity and the quality as a function of the number of carbons employed in the alcohol. The main interest of this study is to give the basis of an efficient synthesis process to produce CNTs of few walls for applications where small diameter is required.

## 1. Introduction

Compared to multi wall carbon nanotubes (MWCNT), production of single and few walls nanotubes is still a challenge and, as a consequence, the price of this type of material is around 20 times higher than the multiwall counterpart. This significant difference in the production cost is in part due to the high energy consumption for the available production methods such as HiPco, Thermal Plasma Synthesis, Arc Discharge Process, *etc.* [1,2,3] and the low production rate of these processes. For material reinforcement, sensor developments, and other applications, CNTs of small diameter and high surface science are required, however SWCNTs could be substituted by CNTs of similar morphology, with few walls, with the advantage of a significant lower price.

Previous MWCNT synthesis research [4,5] showed that under the presence of heterogeneous atoms, different from carbon and hydrogen, the morphological characteristics of produced CNTs differ greatly from the ones obtained from sources like benzene, toluene or xylene. Thus, we study the use of aliphatic alcohols as the carbon source despite the presence of oxygen on the precursor molecule, which could suggests that under temperature of synthesis, some oxidation is possible during the synthesis, with the consequent formation of CO, CO_2_, iron oxides and/or structural deformities and so, lower yield.

Some reports have mentioned the use of alcohols as carbon source for the CNTs and have completed a systematic study of the product regarding the effect of the alcohol carbon length, catalysts used and/or changes with the CVD method [6]. In a recent work by Bystrzejewski *et al.*, they mention the use of alcohols ranging from methanol to decanol, where they obtained single walled nanotubes (SWNT) from alcohols of one to six carbon atoms and multiwall nanotubes (MWNT) with longer chain alcohols, stating that carbon chains in alcohol molecules influence the crystallinity of the product [6]. Nevertheless, the reported synthesis implies a low rate production method, since it takes around three hours to produce quantities between 200 and 400 mg of product. Moreover, other investigations showed that the addition of different types of precursors as nitrogen into the aliphatic alcohol played an important role in modifying both the CNT growth and morphology [7,8]. 

Other studies mention the importance of using different catalysts like Fe–Co [9,10], Fe–Ni [11,12] or Fe–Mo [13], as a way to influence the growth on zeolite (MgO) supported CNTs [14,15]. However, in all these articles, at least two steps are required: the formation of catalyst nanoparticles over substrate particles, followed by the CNTs synthesis under a carbon source flow with the consequent manufacturing delay.

Recent studies on synthesis by nebulized spray pyrolysis report negative effects on the CNTs production yield when the carbon source (toluene or benzene) is mixed with alcohols [16,17], probably acquainted to the formation of radicals during the nebulization step. Additional results claim that the influence over the growth characteristics of CNTs using aliphatic alcohols depends of the OH radicals [17,18,19].

**Figure 1:**
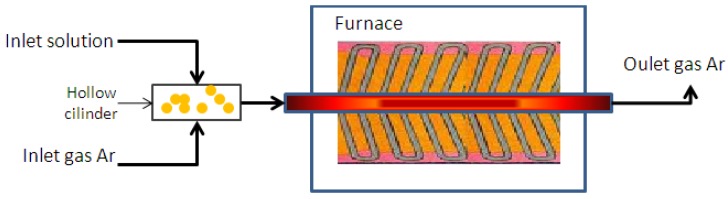
Schematic diagram of reaction system for the synthesis of CNTs by spray pyrolysis.

The alternative spray pyrolysis method used in this work consists in the introduction of the precursor into a stainless steel pre-heater maintained at constant temperature (Figure 1) with the purpose of changing the liquid precursors to their vapor phases before entering the reaction chamber where the CNTs are grown up. Using this technique we anticipate the establishment of a new base that allows producing small diameter CNTs close to the reported SWCNTs with the advantage of low cost and simple process. 

## 2. Results and Discussion

**Figure 2:**
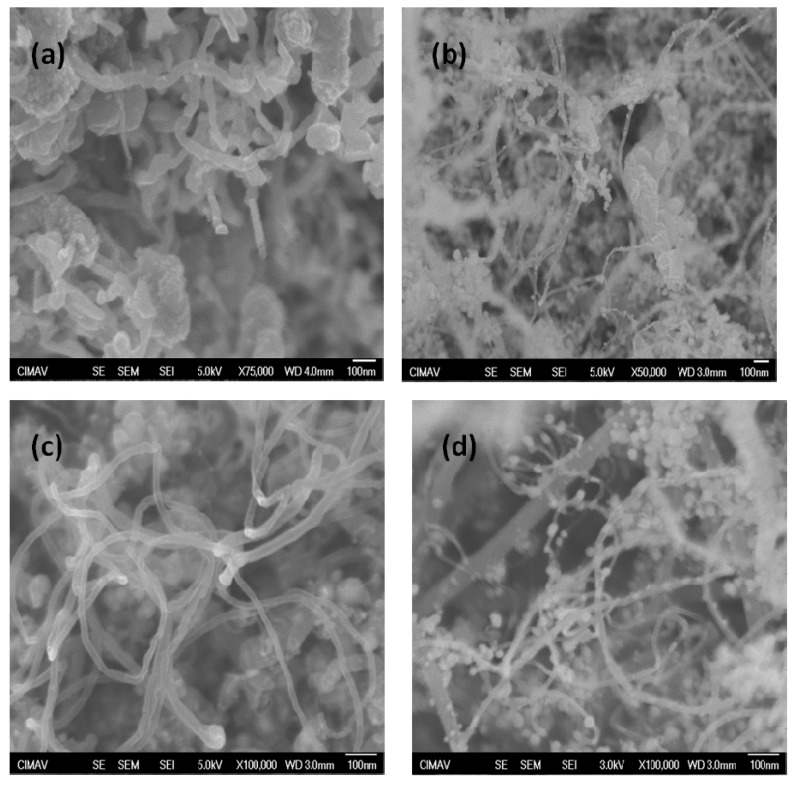
SEM images of carbon nanotubes obtained with the different carbon sources: (**a**) Methanol; (**b**) Ethanol; (**c**) Propanol and (**d**) Butanol.

Figure 2 shows SEM images that reveal the variation of morphology of CNTs as a function of the alcohol used as carbon source. As can be observed, impurities are different in size and shape for the samples. Energy dispersive X-ray spectrometry (EDS) analysis shows the presence of iron nanoparticles, which is formed from the ferrocene. As is well known, ferrocene is employed as a catalyst to promote the formation of graphene sheets, to conform CNTs walls during the synthesis process. Typically, the quantity collected of product was on the average 0.65 g for methanol, 0.30 g for ethanol and 0.35 g for propanol and butanol. On the other hand, a higher ratio of CNTs/amorphous carbon was obtained for propanol precursor. Amorphous carbon has been found mainly with an onion shape and an iron nanoparticle core. 

From the SEM micrographs, diameters and lengths of the CNTs were determined. Average values of these parameters were obtained by measuring length and diameter of the most representative nanotubes from each sample, and results are summarized in Table 1. It is observed that the average diameter varies inversely to the number of carbon atoms contained in the precursor alcohol molecule, however there is not a significant difference for propanol and butanol. Furthermore, average length is directly proportional to the number of carbon atoms. 

**Table 1:** Average external diameters, lengths and number of walls of carbon nanotubes.

alcohol	average external diameter	average length	average number of walls
	nm	μm	
Methanol (CH_4_O)	24	0.5	35
Ethanol (C_2_H_6_O)	18	1.2	10
Propanol (C_3_H_8_O)	15	2.0	8
Butanol (C_4_H_10_O)	16	2.2	6

According to previous work [4], before the deposition of carbon/iron precursors on substrate in the gaseous phase, there is a thermal cracking of the carbon source molecule, which under the presence of ferrocene leads to the reduction of iron and its coalescence to form nanoparticles and, in this case, it is on the surface of those nanoparticles that the formation of graphene walls from alcohol precursor is carried out. 

Nanotubes obtained from methanol have larger outer diameters and a shorter length, and most of the product consists of onion shape structures, which means that for this specific method of CNTs synthesis, methanol has higher trend to surround iron nanoparticles instead of the promotion of the formation of the cylinder structures required to obtain large CNTs. The CNTs obtained from butanol nanotubes show the best CTNs/amorphous carbon ratio, thus this molecule seems to have the most adequate structure to promote the formation of basic C_6_ rings, and the formation of the cylinder structures required to generate the nanotubes. 

**Figure 3:**
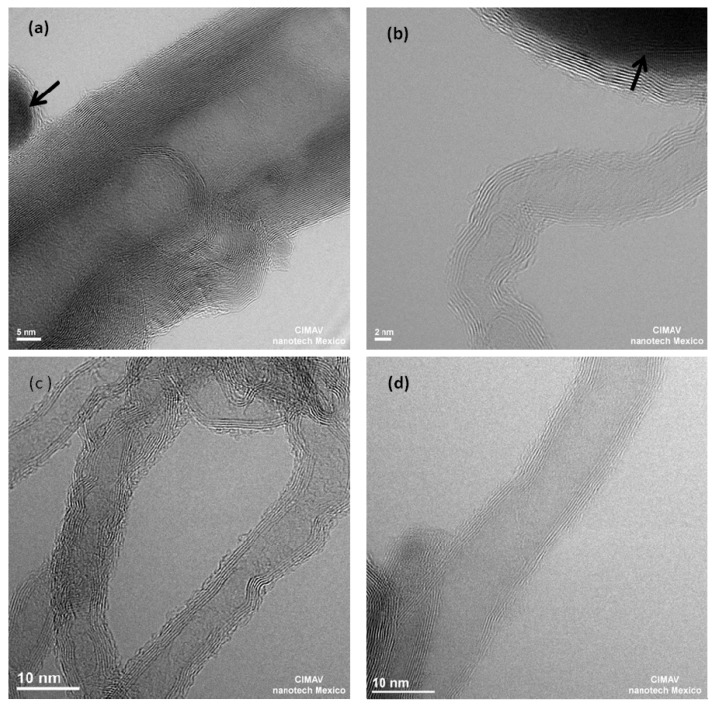
TEM images of carbon nanotubes obtained from: (**a**) Methanol; (**b**) Ethanol; (**c**) Propanol and (**d**) Butanol. Arrows indicate iron nanoparticles and carbon encapsulated structures.

The number of layers that constitute the nanotubes walls, are obtained from the HRTEM micrographs, as shown in Figure 3, where it can be seen how the iron nanoparticles (identified with an arrow) promotes the formation of graphite walls that constitute the CNTs. The size of the iron particles was found in the range 10–40 nm. However, considering the CNTs formation mechanism, the use of alcohols as precursors difficult the organization of the graphite wall with the consequence of having CNTs with higher number of structural defects.

The methanol sample (Figure 3a), shows that the number of walls that constitute these CNTs is typically of the order of 35. In the ethanol case (Figure 3b), the iron nanoparticles are bigger than the nanoparticles in the other precursors and the number of walls diminishes to about 10.

Figure 3c,d show HRTEM micrographs from propanol- and butanol-based CNT, respectively. It is observed that the average numbers of nanotube walls are similar, around 8 for propanol and 6 for butanol. These results are summarized in Table 1, where it is clearly observed how the number of layers in the CNTs decreases as the number of carbon atoms in the precursor molecule increases. 

**Figure 4:**
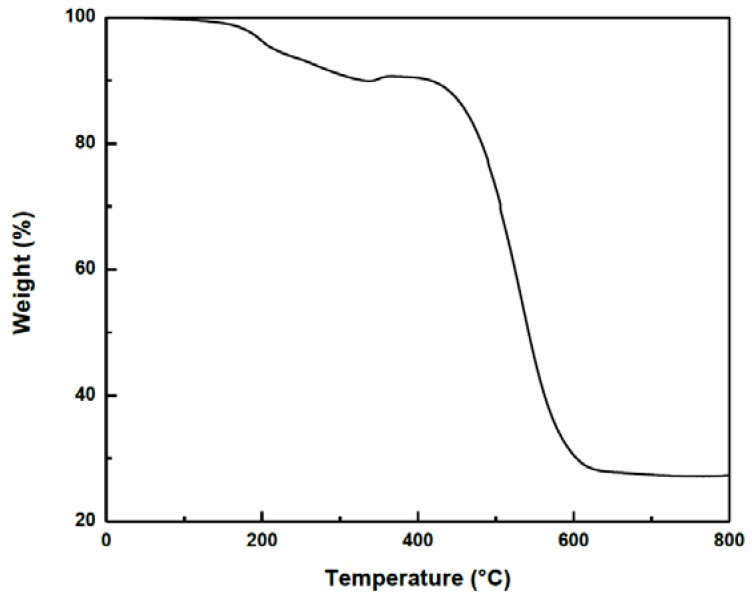
Raman spectra of propanol-based CNTs using laser excitation at E_laser_ = 632.8 nm.

In order to discuss the quality of the product, we performed Thermogravimetric Analysis (TGA) and Raman spectroscopy. Figure 4 shows the TGA profile of the propanol-based sample. The TGA reveals that the mass loss occurs in three steps. At T < 200 °C, there is a mass loss of about 5% that can be attributed to the evaporation of heavy organic material introduced at the end of the synthesis process, just before the rapid cooling of the tube. In the range 200–400 °C we have a mass loss from the evaporation of amorphous carbon. Based on these results, we calculate about 10% organic impurities in the sample. The residual mass, attributed to ferric oxide has been estimated at 27% and coming from metallic iron (19%) in the nanotubes, oxidized during the analysis. Accordingly, the CNTs evaporated in the temperature range 200–400 °C represents 71% of mass contents in the sample. This means that the ratios of amorphous carbon/CNTs and iron/CNTs are 0.07 and 0.27, respectively. 

**Figure 5:**
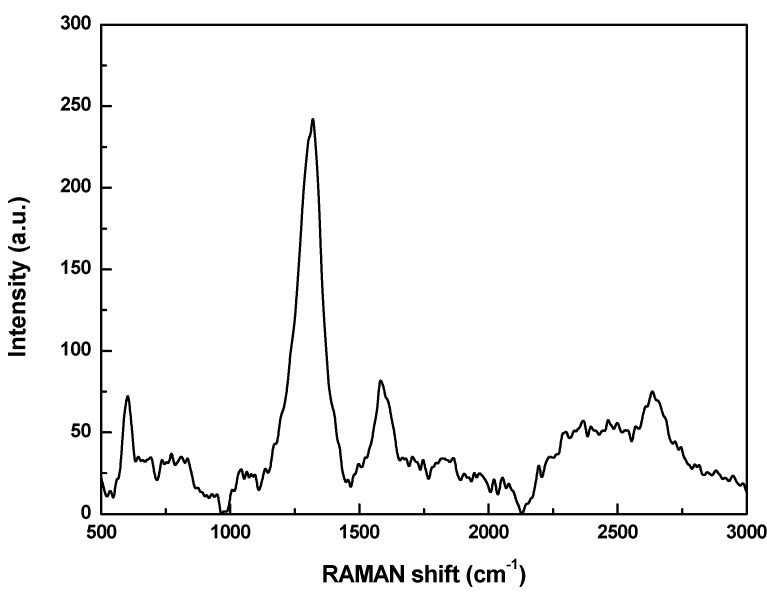
Thermogravimetry analysis (TGA) analysis of propanol-based CNT.

Figure 5 shows the Raman shift spectrum from propanol-based sample. The two main typical graphite bands are present in the Raman spectrum of the nanotubes: the band at 1582 cm^−1^ (G band) assigned to the in-plane vibration of the C–C bond (G band) typical of defective graphite-like materials and the band between at 1320 cm^−1^ (D band) activated by the presence of disorder in carbon systems. The Raman spectrum also exhibits a band at 2635 cm^−1^ (G′ band) attributed to the overtone of the D band. These bands have been obtained on the as-received powder of MWCNTs. We found that the relative intensity I_D_/I_G_ is constant for all samples, indicating that the reaction does not modify the crystalline structure of the material. According to the literature [20,21,22,23,24], samples containing large amounts of MWCNTs, display higher D-bands than samples containing small amounts, which is characteristic of the employed laser energy (638 nm). However, some authors suggest that MWCNTs tend to appear when there is a higher degree of defects in the nanotubes [25,26,27].

## 3. Experimental

A liquid solution of ferrocene and alcohol were mixed with argon gas in a preheated stainless steel cylinder that converts this mix into vapor. Temperature of 180 °C in the preheater was found optimal for the aliphatic alcohols to reach its vapor phase [28,29,30,31]. Afterwards, this vapor is injected through a silicon hose into a quartz substrate tube placed inside a horizontal furnace set at a temperature of 750 °C where the CNTs grow up. Ferrocene/alcohol concentration used in the synthesis was set to 0.12 g/25 mL, inner and outer work tube diameters were 9 and 11 mm, respectively and the length of the furnace is 350 mm.

The alcohol/ferrocene solution was injected into the preheated stainless steel cylinder at a rate of 1 mL/min during 20 min, with the help of a dispenser. The vapor obtained was carried to the reactor by dry argon flow at 0.32 L/min. After the solution is dispensed, the reactor is turned off and let it to cool below a temperature of 400 °C. The whole procedure takes about 25 min to complete. 

Various syntheses were performed at different temperatures, ranging from 700 to 900 °C in 50 °C steps. We observed that at 700 °C the sample showed almost no carbon nanotubes inside the quartz tube, and from 850 to 900 °C we found the presence of graphite. Experiments show that the best nanotubes quality and quantity are obtained for temperatures in the range 750–800 °C.

The mass of ferrocene in the synthesis process was optimized, by using several amounts, 0.20, 0.25 and 0.30 g of ferrocene. The sample obtained with 0.25 g showed a smaller amount of iron impurities than the sample with 0.30 g and a larger amount of nanotubes production than the sample with 0.20 g.

Morphology and microstructural characterization of the CNTs were performed by scanning electron microscopy (SEM) in a JSM-7401F instrument operated at 3–5 kV and by high resolution transmission electron microscopy (HRTEM) in a JEOL JEM-2100FS with beam Cs-corrector, operated at 200 kV, with a spatial resolution close to 0.13 nm.

The quality of the products was determined by thermogravimetry analysis (TGA), using a TA Instruments Q600 thermal analyzer at a heating rate of 3 °C/min in air and Raman spectra were acquired with a LabRam Horiba HR system, using a 632.8 nm He-Ne laser at 14.2 mW, equipped with a column of CCD detectors cooled at −75 °C. The resolution obtained is about 1 cm^−1^.

## 4. Conclusions 

A spray pyrolysis technique to produce MWNTs of only few walls, through the use of aliphatic alcohols was presented. In particular we report the successful production of CNTs using this method with methanol, ethanol, propanol and butanol. Furthermore, we demonstrate that the number of carbons on the alcohol employed has a strong influence in the number of walls of the synthesized CNTs and the CNTs/amorphous carbon ratio, as can be inferred from the structural analysis carried out by scanning electron microscopy (SEM) and transmission electron microscopy (TEM). 
